# A New Data Mining Scheme Using Artificial Neural Networks

**DOI:** 10.3390/s110504622

**Published:** 2011-04-28

**Authors:** S. M. Kamruzzaman, A. M. Jehad Sarkar

**Affiliations:** 1 Department of Electronics Engineering, Hankuk University of Foreign Studies, 89 Wangsan-ri, Mohyeon-myon, Yongin-si, Kyonggi-do, 449-791, Korea; 2 Department of Digital Information Engineering, Hankuk University of Foreign Studies, 89 Wangsan-ri, Mohyeon-myon, Yongin-si, Kyonggi-do, 449-791, Korea; E-Mail: jehad@hufs.ac.kr

**Keywords:** data mining, neural networks, symbolic rules, weight freezing, constructive algorithm, pruning, clustering, rule extraction, symbolic rules

## Abstract

Classification is one of the data mining problems receiving enormous attention in the database community. Although artificial neural networks (ANNs) have been successfully applied in a wide range of machine learning applications, they are however often regarded as black boxes, *i.e.*, their predictions cannot be explained. To enhance the explanation of ANNs, a novel algorithm to extract symbolic rules from ANNs has been proposed in this paper. ANN methods have not been effectively utilized for data mining tasks because how the classifications were made is not explicitly stated as symbolic rules that are suitable for verification or interpretation by human experts. With the proposed approach, concise symbolic rules with high accuracy, that are easily explainable, can be extracted from the trained ANNs. Extracted rules are comparable with other methods in terms of number of rules, average number of conditions for a rule, and the accuracy. The effectiveness of the proposed approach is clearly demonstrated by the experimental results on a set of benchmark data mining classification problems.

## 1. Introduction

Data mining, also popularly known as knowledge discovery in databases, refers to the process of automated extraction of hidden, previously unknown and potentially useful information from large databases. It is the process of finding and interpreting the valuable information by using the knowledge of multidisciplinary fields such as statistics, artificial intelligence, machine learning, database management and so on [1,2]. While the predictive accuracy obtained by artificial neural networks (ANNs) is often higher than that of other methods or human experts, it is generally difficult to understand how ANNs arrive at a particular conclusion due to the complexity of the ANN architectures [3,4]. It is often said that an ANN is practically a “black box”. Even for an ANN with only single hidden layer, it is generally impossible to explain why a particular pattern is classified as a member of one class and another pattern as a member of another class, due to the complexity of the network [5]. This may cause problems in some cases. To solve this problem, researchers are interested in developing a humanly understandable representation for ANNs.

ANNs have the ability of distributed information storage, parallel processing, reasoning, and self-organization. It also has the capability of rapid fitting of nonlinear data, so it can solve many problems which are difficult for other methods [6]. Initially, the application of the ANN in data mining was not positive, and the main reasons were that the ANN has the defects of complex structure, poor interpretability and long training times. But its advantages such as high affordability to the noise data with low error rate, and the continuously advancing and optimization of various network training, pruning, and rule extraction algorithms, make the application of the ANNs in the data mining increasingly favored by the overwhelming majority of users [7–9]. In machine learning and data mining research, rule extraction has become an increasingly important topic, and a growing number of researchers and practitioners have applied ANNs for machine learning in a variety of real world applications [10–14]. An inherent defect of ANNs is that the learned knowledge is masked in a large amount of connections, which leads to the poor transparency of knowledge and poor explanation ability [15]. In order to compensate this defect, developing algorithms to extract symbolic rules from trained neural networks has been a hot topic in recent years.

In many applications, it is highly desirable to extract symbolic rules from these networks. Unlike a collection of weights, symbolic rules can be easily interpreted and verified by human experts. They can also provide new insights into the application problems and the corresponding data [16,17]. A number of works are available in the literature to explain the functionality of ANNs by extracting rules from trained ANNs. The main problem of existing works is that they determine the number of hidden neurons in ANNs manually. Thus, the prediction accuracy and rules extracted from trained ANNs may not be optimal since the performance of ANNs is greatly dependent on their architectures. Furthermore, rules extracted by existing algorithms are not simple; as a result it is difficult to understand by the users.

In this paper we have proposed a new data mining scheme; referred to as ESRNN (Extraction of Symbolic Rules from ANNs) to extract symbolic rules from trained ANNs. A four-phase training algorithm is proposed by using backpropagation learning. In the first and second phases, appropriate network architecture is determined using weight freezing based constructive and pruning algorithms. In the third phase, the continuous activation values of the hidden nodes are discretized by using an efficient heuristic clustering algorithm. Finally, in the fourth phase, symbolic rules are extracted using the frequently occurred pattern based rule extraction algorithm by examining the discretized activation values of the hidden nodes.

The rest of the paper is organized as follows. Section 2 describes the related work. The proposed data mining scheme is presented in Section 3. We discuss the performance evaluation in Section 4. Finally, in Section 5 we conclude the paper.

## 2. Related Work

A neural network-based approach to mining classification rules from given databases has been proposed in [18]. The network is first trained to achieve some required accuracy rate. Redundant connections of the network are then removed by a network pruning algorithm. The activation values of the hidden nodes in the network are analyzed, and classification rules are generated using the result of this analysis. Two classes of approaches for data mining with ANNs have been proposed in [19]. The first approach, often called rule extraction, involves extracting symbolic models from trained neural networks. The second approach is to directly learn simple, easy-to-understand networks. Data mining using pruned artificial neural network tree (ANNT) has been proposed in [20]. ANNT pruning approach consists of three phases: training, pruning and rule extraction. It improved the generalization ability of the network and the number of rules extracted is reduced. The key technology and ways to achieve the data mining based on neural networks is researched in [7]. The combination of data mining method and neural network model can greatly improve the efficiency of data mining techniques, and has been widely used. How to apply ANN in data mining techniques has reviewed in [2]. Given the current state of the art, neural network deserves a place in the tool boxes of data mining specialists.

In the literature, there are many different approaches for the rule extraction from ANNs. A number of algorithms for extracting rules from trained ANNs have been developed in the last two decades [21–30]. Saito and Nakano proposed a medical diagnosis expert system based on a multilayer ANN in [21]. They treated the network as a black box and used it only to observe the effects on the network output caused by change the inputs. Two methods for extracting rules from ANN are described by Towell and Shavlik in [22]. The first method is the subset algorithm [23], which searches for subsets of connections to a node whose summed weight exceeds the bias of that node. The major problem with subset algorithms is that the cost of finding all subsets increases as the size of the ANNs increases. The second method, the MofN algorithm [24], is an improvement of the subset method that is designed to explicitly search for M-of-N rules from knowledge based ANNs. Instead of considering an ANN connection, groups of connections are checked for their contribution to the activation of a node, which is done by clustering the ANN connections.

Liu and Tan proposed X2R in [25], a simple and fast algorithm that can be applied to both numeric and discrete data, and generate rules from datasets. It can generate perfect rules in the sense that the error rate of the rules is not worse than the inconsistency rate found in the original data. The problem of the rules generated by X2R, are order sensitive, *i.e.*, the rules should be fired in sequence. Liu described a family of rule generators in [26] that can be used to extract rules in various applications. It includes versions that can handle noise in data, produce perfect rules, and can induce order independent or dependent rules. The basic idea of the algorithm is simple: using first order information in the data to determine shortest sufficient conditions in a pattern that can differentiate the pattern from patterns belonging to other classes.

Setiono presented MofN3, a new method for extracting M-of-N rules from ANNs, in [27]. The topology of the ANN is the standard three-layered feedforward network. Nodes in the input layer are connected only to the nodes in the hidden layer, while nodes in the hidden layer are also connected to nodes in the output layer. Given a hidden node of a trained ANN with *N* incoming connections, show how the value of *M* can be easily computed. In order to facilitate the process of extracting M-of-N rules, the attributes of the dataset have binary values −1 or 1. Kamruzzaman and Islam proposed an algorithm, REANN in [28] to extract rules from trained ANNs for medical diagnosis problems. This paper investigates the rule extraction process for only 3 medical datasets.

Jin and Sendhoff provide an up-to-date yet not necessarily complete review of the existing research on Pareto-based multiobjective machine learning (PMML) algorithms in [29]. They illustrate, on three benchmark problems (breast cancer, iris, and diabetes), how can address important topics in machine learning, such as generating interpretable models, model selection for generalization, and ensemble extraction, using the Pareto-based multiobjective approach. They compare three Pareto-based approaches to the extraction of neural ensembles and indicate that the method by trading off accuracy and complexity can provide reliable results. Finally, Wang *et al.* proposed a novel algorithm of regression rules extraction from ANN in [30], which is based on linear intelligent insertion. The linear function and symbolic rules are used to the ANN, and the rules are generated by the decision tree.

The limitations of the existing rule extraction algorithms are summarized as follows:

- Use predefined and fixed number of hidden nodes that require human experience and prior knowledge of the problem to be solved,
- Clustering algorithms used to discretize the output values of hidden nodes are not efficient,
- Computationally expensive,
- Could not produce concise rules, and
- Extracted rules are order sensitive.

To overcome these limitations we have proposed a scheme for data mining by extracting symbolic rules from trained ANNs. The proposed system successfully solves a number of data mining classification problems in the literature and described in detail in the next section.

## 3. Proposed Data Mining Scheme Using ANNs

**Figure 1.**
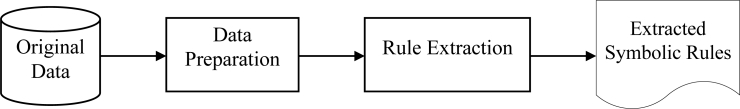
Data mining technique using ANNs.

Developing algorithms and applications that are able to gain knowledge of their experience and previous examples, and that show intelligent behavior is the domain of machine learning and ANNs. Data mining on the other hand deals with the analysis of large and complex databases in order to discover new, useful and interesting knowledge using techniques from machine learning and statistics. The data mining process using ANNs with the emphasis on symbolic rule extraction is described in this section. The proposed data mining scheme is composed of two steps: data preparation and rule extraction, as shown in Figure 1 and explained further as follows:

### 3.1. Data Preparation

In many fields of artificial intelligence, such as pattern recognition, information retrieval, machine learning, and data mining, one needs to prepare quality data by pre-processing the raw data. The input to the data mining algorithms is assumed to be nicely distributed, containing no missing or incorrect values where all features are important. The real-world data may be incomplete, noisy, and inconsistent, which can disguise useful patterns. Data preparation is a process of the original data to make it fit to a specific data mining method. Data preparation is the first important step in the data mining and plays an important role in the entire data mining process.

The data mining using ANNs can only handle numerical data. How to represent the input and output attributes of a learning problem in a neural network is one of the key decisions influencing the quality of the solutions one can obtain. Depending on the kind of problem, there may be several different kinds of attributes that must be represented. For all of these attribute kinds, multiple reasonable methods of neural network representation exist. We will now discuss each attribute kind and some common methods to represent such an attribute.

- **Real-valued attributes** are usually rescaled by some function that maps the value into the range 0…1 or −1…1 in a way that makes a roughly even distribution within that range.
- **Integer-valued attributes** are most often handled as if they were real-valued. If the number of different values is only small, one of the representations used for ordinal attributes may also be appropriate. Note that often attributes whose values are integer numbers are not really integer-valued but are ordinal or cardinal instead. We consider all integer-valued attributes as real-valued.
- **Ordinal attributes** with *m* different values are either mapped onto an equidistant scale making them pseudo-real-valued or are represented by *m* −1 inputs of which the leftmost *k* have value 1 to represent the *k*-th attribute value while all others are 0. A binary code using only [log_2_ *m*] inputs can also be used.
- **Nominal attributes** with *m* different values are usually either represented using a 1-of-*m* code or a binary code.
- **Missing attribute** values can be replaced by a fixed value (e.g., the mean of the non-missing values of this attribute) or can be represented explicitly by adding another input for the attribute that is 1 if the attribute value is missing.

### 3.2. Rule Extraction: The ESRNN Algorithm

It is becoming increasingly apparent that without some form of explanation capability, the full potential of ANNs may not be realized. The rapid and successful proliferation of applications incorporating ANNs in many fields, such as commerce, science, industry, medicine *etc.*, offers a clear testament to the capability of ANN paradigm. Extracting symbolic rules from trained ANN is one of the promising areas that are commonly used to explain the functionality of ANNs. The aim of this subsection is to introduce a new algorithm, referred to as ESRNN (extraction of symbolic rules from ANNs), to extract symbolic rules from trained ANNs. We now describe below each of the components of ESRNN in further detail.

**Figure 2.**
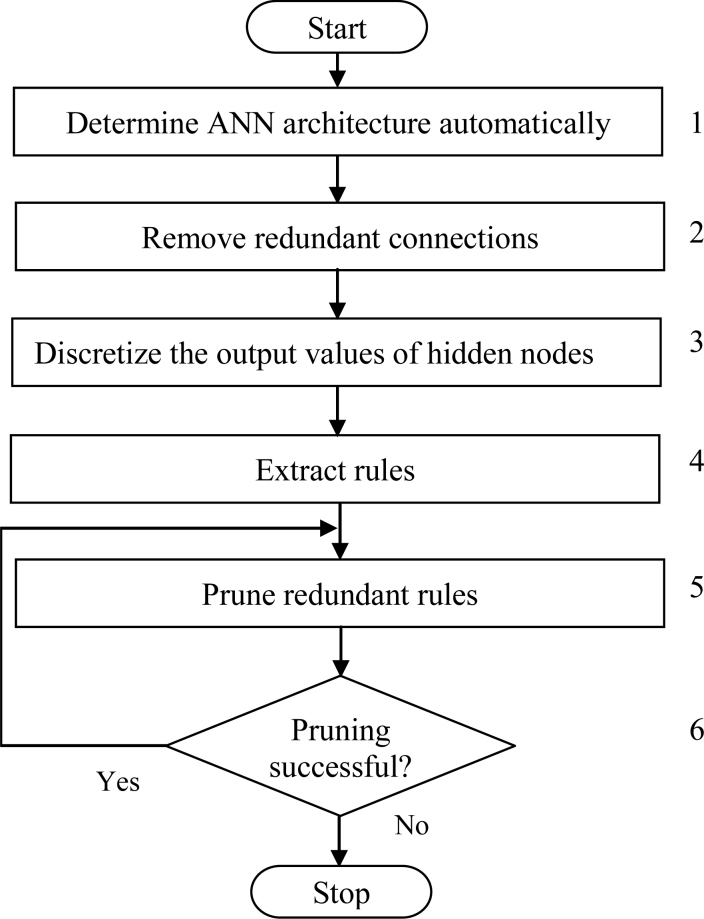
Flow chart of the proposed ESRNN algorithm.

A standard three-layer feedforward ANN is the basis of the proposed ESRNN algorithm. The hyperbolic tangent function, which can take any value in the interval [–1, 1], is used as the hidden node activation function. Rules are extracted from near optimal ANN by using a new rule extraction algorithm. The aim of ESRNN is to search for simple rules with high predictive accuracy. The major steps of ESRNN are summarized in Figure 2 and explained further as follows:

**Step 1** Create an initial ANN architecture. The initial architecture has three layers, including an input, an output, and a hidden layer. The number of nodes in the input and output layers is the same as the number of attributes and the classes of the problem. Initially, the hidden layer contains only one node. The number of nodes in the hidden layer is automatically determined by using the weight freezing based constructive algorithm, explained in subsection *A.* Initialize all connection weights randomly within a certain small range.
**Step 2** Remove redundant input nodes and connections between input nodes and hidden nodes and between hidden nodes and output nodes by using a basic pruning algorithm, explained in subsection *B*. When pruning is completed, the ANN architecture contains only important nodes and connections. This architecture is saved for the next step.
**Step 3** Discretize the outputs of hidden nodes by using an efficient heuristic clustering algorithm, explained in subsection *C*. The reason for discretization is that the outputs of hidden nodes are continuous, and thus the rules can not be readily extractable from the ANN.
**Step 4** Extract the rules that map the inputs and outputs relationships. The task of the rule extraction is accomplished in three phases. In the first phase, rules are extracted by using the rule extraction algorithm, explained in subsection *D*, to describe the outputs of ANN in terms of the discretized output values of the hidden nodes. In the second phase, rules are extracted to describe the discretized output values of the hidden nodes in terms of the inputs. Finally in the third phase, combine the rules extracted in the first and second phases.
**Step 5** Prune redundant rules extracted in **Step 4** by replacing specific rules with more general ones.
**Step 6** Check the classification accuracy of the network. If the accuracy falls below an acceptable level, *i.e.*, rule pruning is not successful then stop. Otherwise go to **Step 5**.

The rules extracted by ESRNN are compact and comprehensible, and do not involve any weight values. The accuracy of the rules from pruned networks is as high as the accuracy of the original networks. The important features of the ESRNN algorithm are the rules extracted by rule extraction algorithm is recursive in nature and is order insensitive, *i.e.*, the rules need not to be required to fire sequentially.

#### 3.2.1. Weight Freezing Based Constructive Algorithm

One drawback of the traditional backpropagation algorithm is the need to determine the number of nodes in the hidden layer prior to training. To overcome this difficulty, many algorithms that construct a network dynamically have been proposed in [31–33]. The most well known constructive algorithms are dynamic node creation (DNC) [34], feedforward neural network construction (FNNC) algorithm, and the cascade correlation (CC) algorithm [35]. The constructive algorithm used in the ESRNN algorithm is based on the FNNC algorithm proposed in [36]. In FNNC algorithm, the training process is stopped when the classification accuracy on the training set is 100% [37]. However, it is not possible to get 100% classification accuracy for most of the benchmark classification problems. In addition, higher classification accuracy on the training set does not guarantee the higher generalization ability *i.e.*, classification accuracy on the testing set.

The training time is an important issue in designing ANNs. One approach for reducing the number of weights to be trained is to train few weights rather than all weights in a network and keep remaining weights fixed, commonly known as weight freezing. The idea behind the weight freezing-based constructive algorithm is to freeze input weights of a hidden node when its output does not change much in the successive few training epochs. Theoretical and experimental studies reveal that some hidden nodes of an ANN maintain almost constant output after some training epochs, while others continuously change during the whole training period.

**Figure 3.**
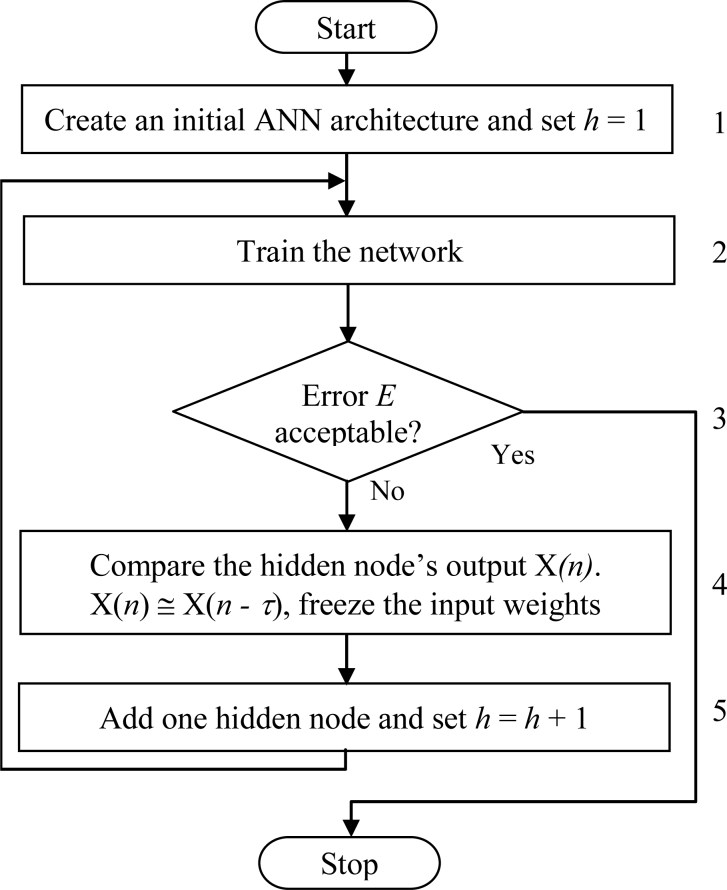
Flow chart of the weight freezing based constructive algorithm.

In our algorithm, it has been proposed that the output of a hidden node can be frozen when its output does not change much in the successive training epochs. This weight freezing method can be considered as combination of the two extremes: for training all the weights of ANNs and for training the weights of only the newly added hidden node of ANNs [38]. The major steps of our weight freezing based constructive algorithm are summarized in Figure 3 and explained further as follows:

**Step 1** Create an initial ANN consisting of three layers, *i.e.*, an input, an output, and a hidden layer. The number of nodes in the input and output layers is the same as the number of inputs and outputs of the problem. Initially, the hidden layer contains only one node *i.e.*, *h* = 1, where *h* is the number of hidden nodes in the network. Randomly initialize all connection weights within a certain range.
**Step 2** Train the network on the training set by using backpropagation algorithm until the error *E* is almost constant for a certain number of training epochs, *τ*, is specified by the user.
**Step 3** Compute the ANN error *E*. If *E* is found unacceptable (*i.e.*, too large), then assume that the ANN has inappropriate architecture, and go to the next step. Otherwise stop the training process. The ANN error *E* is calculated according to the following equations:
  (1) $$E(w,\;v)=\frac{1}{2}\sum_{i=1}^{k}\sum_{p=1}^{C}{\left(S_{pi}-t_{pi}\right)}^{2}$$
  where *k* is the number of patterns, *C* is the number of output nodes, and *t_pi_* is the target value for pattern *x_i_* at output node *p. S_pi_* is the output of the network at output node *p*.
  (2) $$S_{pi}=\sigma (\sum_{m=1}^{h}\delta ({\left(x_{i}\right)}^{T}\;w_{m})v_{pm})$$
  where *h* is the number of hidden nodes in the network, *x_i_* is an *n*-dimensional input pattern, *i* = 1, 2, …, *k*, *w_m_* is a *p*-dimensional vector weights for the arcs connecting the input layer and the *m*-th hidden node, *m* = 1, 2, …, *h*, *v_m_* is a *C*-dimensional vector of weights for the arcs connecting the *m*-th hidden node and the output layer. The activation function for the output layer is sigmoid function *σ*(*y*) = 1/ (1 + *e*^−*y*^) and for the hidden layer is hyperbolic tangent function *δ*(*y*) = (*e^y^* − *e*^−*y*^) / (*e^y^* + *e*^−*y*^).
**Step 4** Compare each hidden node’s output *X* (*n*) at training epoch *n* with its previous value *X* (*n* − *τ*). If *X* (*n*) ≅ *X* (*n* − *τ*), freeze the input weights of that node.
**Step 5** Add one hidden node to the hidden layer. Randomly initialize the weights of the arcs connecting this new hidden node with input nodes and output nodes. Set *h* = *h* + 1 and go to **Step 2**.

#### 3.2.2. Pruning Algorithm

Pruning offers an approach for dynamically determining an appropriate network topology. Pruning techniques begin by training a larger than necessary network and then eliminate weights and nodes that are deemed redundant [38,39].

As the nodes of the hidden layer are determined automatically by weight freezing based constructive algorithm in ESRNN, the aim of this pruning algorithm used here is to remove as many unnecessary nodes and connections as possible. A node is pruned if all the connections to and from the node are pruned. Typically, methods for removing weights from the network involve adding a penalty term to the error function. It is hoped that by adding a penalty term to the error function, unnecessary connections will have small weights, and therefore pruning can reduce the complexity of the network significantly. The simplest and most commonly used penalty term is the sum of the squared weights.

Given a set of input patterns *x_i_* ∈ ℜ*^n^*, *i* = 1, 2, …, *k*, let *w_m_* is a *p*-dimensional vector weights for the arcs connecting the input layer and the *m*-th hidden node, *m* = 1, 2, …, *h*. The weight of the connection from the *l*-th input node to the *m*-th hidden node is denoted by *w_ml_*, *v_m_* is a *C*-dimensional vector of weights for the arcs connecting the *m*-th hidden node and the output layer. The weight of the connection from the *m*-th hidden node to the *p*-th output node is denoted by *v_pm._* It has been suggested that faster convergence can be achieved by minimizing the cross entropy function instead of squared error function [40].

The backpropagation algorithm is applied to update the weights (*w*, *v*) and minimize the following error function:

(3) θ(w, v)=F(w, v)+P(w, v)

where *F*(*w*, *v*) is the cross entropy function:

(4) $$F(w,\;v)=-\sum_{i=1}^{k}\sum_{p=1}^{o}\left(t_{pi}\;\text{log}\;S_{pi}+(1-t_{pi})\;\text{log}(1-S_{pi})\right)$$

where *S_pi_* is the output of the network:

(5) $$S_{pi}=\sigma (\sum_{m=1}^{h}\delta ({\left(x_{i}\right)}^{T}\;w_{m})v_{pm})$$

where *x_i_* is an *n*-dimensional input pattern, *i* = 1, 2, …, *k*, and (*x_i_*)*^T^* *w_m_* denotes the scalar product of the vectors *x_i_* and *w_m_*, *δ*(.) is the hyperbolic tangent function and *σ*(.) is the logistic sigmoid function. *P*(*w*, *v*) is a penalty term used for weight decay:

(6) $$P(w,\;v)={ɛ}_{1}\left(\sum_{m=1}^{h}\sum_{l=1}^{n}\frac{\beta {\left(w_{ml}\right)}^{2}}{1+\beta {\left(w_{ml}\right)}^{2}}+\sum_{m=1}^{h}\sum_{p=1}^{o}\frac{\beta {\left(v_{pm}\right)}^{2}}{1+\beta {\left(v_{pm}\right)}^{2}}\right)+{ɛ}_{2}\left(\sum_{m=1}^{h}\sum_{l=1}^{n}{\left(w_{ml}\right)}^{2}+\sum_{m=1}^{h}\sum_{p=1}^{o}{\left(v_{pm}\right)}^{2}\right)$$

The values for the weight decay parameters *ɛ*_1_,*ɛ*_1_ > 0 must be chosen to reflect the relative importance of the accuracy of the network verses its complexity. More weights may be removed from the network at the cost of a decrease in the network accuracy with larger values of these two parameters. They also determine the range of values where the penalty for each weight in the network is approximately equal to *ɛ*_1_. The parameter *β* > 0 determines the steepness of the error function near to the origin.

**Figure 4.**
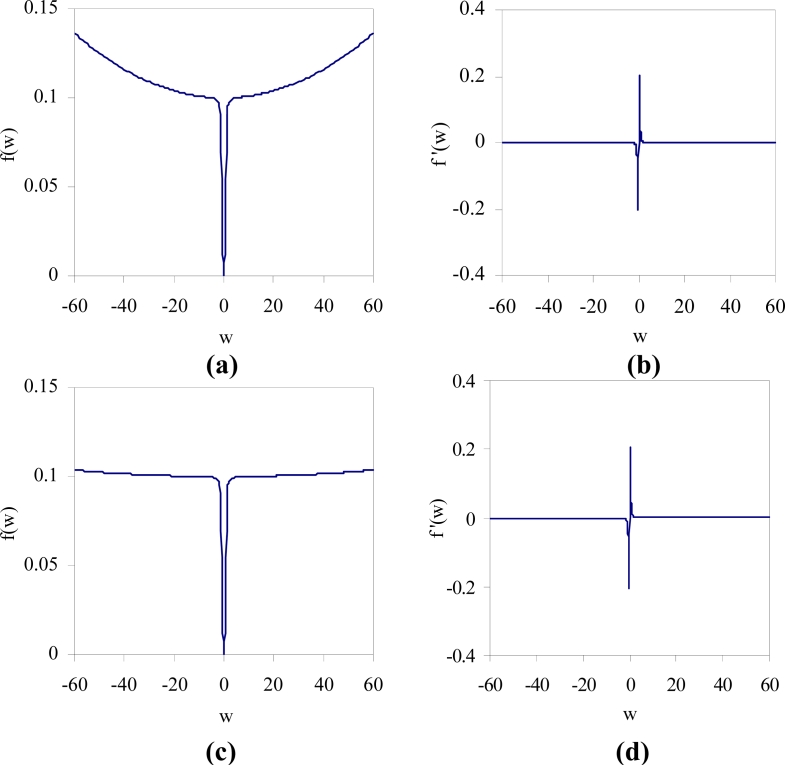
Plots of the function *f* (*w*) = *ɛ*_1_*βw*^2^ / (1 + *βw*^2^) + *ɛ*_2_*w*^2^ and its derivative *f*^′^(*w*) = 2*ɛ*_1_*βw* / (1 + *βw*^2^)^2^ + 2*ɛ*_2_*w*, where *ɛ*_1_ = 0.1, *ɛ*_2_ = 10^−5^, and *β* = 10 for **(a,b)** and *ɛ*_1_ = 0.1, *ɛ*_2_ = 10^−6^, and *β* = 10 for **(c,d)**.

The value of the function *f* (*w*) *w*^2^ / (1 + *w*^2^) is small when *w* is close to zero and approaches to 1 as *w* becomes large. In addition, the derivative function *f*^′^ (*w*) = *w*^2^ / (1 + *w*^2^)^2^ indicates that the backpropagation training will be very little affected with the addition of the penalty function for weights having large values. Consider the plot of the function *f* (*w*) = *ɛ*_1_*βw*^2^ / (1 + *βw*^2^) + *ɛ*_2_*w*^2^ shown in Figure 4, where *ɛ*_1_ = 0.1, *ɛ*_2_ = 10^−5^, and *β* = 10 in Figure 4(a). This function intersects the horizontal line *f* = *ɛ*_1_ at *w* ≈ ±5.62. By decreasing the value of *ɛ*_2_, the interval over which the penalty value is approximately equal to *ɛ*_1_ can be made wider as shown in Figure 4(c), where *ɛ*_2_ = 10^−6^. A weight is prevented from taking too large value, since the quadratic term becomes dominant for the larger values of *w*. The derivative of the function *f* (*w*), *f*^′^ (*w*) near zero is relatively large as shown in Figure 4(b,d). This will give a small weight *w* stronger tendency to decay to zero.

This pruning algorithm removes the connections of the ANN according to the magnitudes of their weights. As the eventual goal of the ESRNN algorithm is to get a set of simple rules that describe the classification process, it is important that all unnecessary nodes and connections must be removed. In order to remove as many connections as possible, the weights of the network must be prevented from taking values that are too large [41]. At the same time, weights of irrelevant connections should be encouraged to converge to zero. The penalty function is found to be particularly suitable for these purposes.

The steps of the pruning algorithm are explained as follows:

**Step 1** Train the network to meet a prespecified accuracy level with the condition (7) satisfied by all correctly classified input patterns.
  (7) $$\underset{p}{\text{max}}|e_{pi}|=\underset{p}{\text{max}}|S_{pi}-t_{pi}|\leq \eta_{1},\;\;p=1,\;2,\ldots ,C.$$
  Let *η*_1_ and *η*_2_ be positive scalars such that (*η*_1_ + *η*_2_) < 0.5 (*η*_1_ is the error tolerance, *η*_2_ is a threshold that determines if a weight can be removed), where *η*_1_ ∈ [0, 0.5). Let (*w*, *v*) be the weights of this network.
**Step 2** Remove connections between input nodes and hidden nodes and between hidden nodes and output nodes. This task is accomplished in two phases. In the first phase, connections between input nodes and hidden nodes are removed. For each *w_ml_* in the network, if
  (8) $$\underset{p}{\text{max}}|v_{pm}\;w_{ml}|\leq 4\eta_{2},$$
  then remove *w_ml_* from the network.
  In the second phase, connections between hidden nodes and output nodes are removed. For each*v_pm_* in the network, if
  (9) $$|v_{pm}|\leq 4\eta_{2}\leq 4\;\eta_{2},$$
  then remove *v_pm_* from the network.
**Step 3** Remove connections between input nodes and hidden nodes further. If no weight satisfies condition (8) or condition (9), then for each *w_ml_* in the network, compute $w_{ml}=\underset{p}{\text{max}}|v_{pm}\;w_{ml}|$. Remove *w_ml_* with smallest *w_ml_*. Continue, otherwise stop.
**Step 4** Retrain the network and calculate the classification accuracy of the network.
**Step 5** If classification accuracy of the network falls below an acceptable level, then stop and use the previous setting of the network weights. Otherwise, go to **Step 2**.

The pruning algorithm used in the ESRNN algorithm intended to reduce the amount of training time. Although it can no longer be guaranteed that the retrained pruned ANN will give the same accuracy rate as the original ANN, the experiments show that many weights can be eliminated simultaneously without deteriorating the performance of the ANN. The two conditions (8) and (9) for pruning depends on the weights for connections between input and hidden nodes and between hidden and output nodes. It is imperative that during the training phase these weights be prevented from getting too large values. At the same time, small weights should be encouraged to decay rapidly to zero.

#### 3.2.3. Heuristic Clustering Algorithm

The process of grouping a set of physical or abstract objects into classes of similar objects is called clustering. A cluster is a collection of data objects that are similar within the same cluster and are dissimilar to the objects in other clusters. A cluster of a data objects can be treated collectively as one group in many applications [42]. There exist a large number of clustering algorithms in the literature, such as, k-means, k-medoids [43,44]. The choice of clustering algorithm depends both on the type of data available and on the particular purpose and applications.

**Figure 5.**
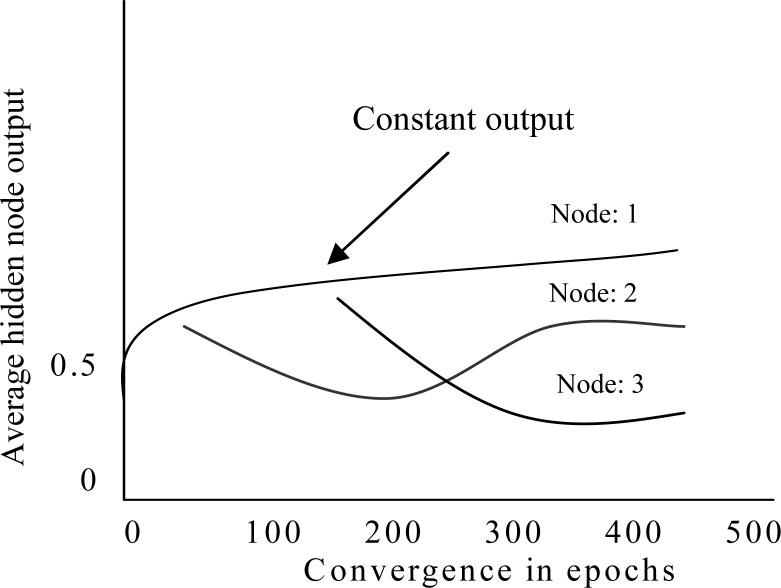
Output of the hidden nodes.

After applying pruning algorithm in ESRNN, the ANN architecture produced by the weight freezing based constructive algorithm contains only important nodes and connections. Nevertheless, rules are not readily extractable because the hidden node activation values are continuous. The discretization of these values paves the way for rule extraction. It is found that some hidden nodes of an ANN maintain almost constant output while other nodes change continuously during the whole training process [45]. Figure 5 shows output of three hidden nodes where a hidden node maintains almost constant output value after some training epochs but output value of other nodes are changing continually. In ESRNN, no clustering algorithm is used when hidden nodes maintain almost constant output value. If the outputs of hidden nodes do not maintain constant value, a heuristic clustering algorithm is used.

The aim of the clustering algorithm is to discretize the output values of the hidden nodes. Consider that the number of hidden nodes in the pruned network is *H*. Clustering the activation values of the hidden node is accomplished by a simple greedy algorithm that can be summarized as follows:

1. Find the smallest positive integer *d* such that if all the network activation values are rounded to *d* decimal places, the network still retains its accuracy rate.
2. Represent each activation value *α* by the integer closest to *α* × 10*^d^*. Let H*_i_* = <*h_i_*_,1_, *h_i,_*_2_, …, *h_i,k_*> be the *k*-dimensional vector of these representations at hidden node *i* for patterns *x*_1_, *x*_2_, …, *x_k_* and let H = (H_1_, H_2_, …, H*_H_*) be the *k* × *H* matrix of the hidden representations of all patterns at all *H* hidden nodes.
3. Let *P* be a permutation of the set {1, 2, …, *H*} and set *m* = 1.
4. Set *i* = *P*(*m*).
5. Sort the values of the *i*th column (H*_i_*) of matrix H in increasing order.
6. Find a pair of district adjacent values *h_i, j_* and *h_i_*_,_ *_j_*_+1_ in H*_i_* such that if *h* *_i_*_,_ *_j_*_+1_ is replaced by *h_i, j_* no conflicting data will be generated.
7. If such a pair of values exists, replace all occurrences of *h* *_i_*_,_ *_j_*_+1_ in H*_i_* by *h_i, j_* and repeat **Step 6**. Otherwise, set *m* = *m* + 1. If *m* ≤ *H*, go to **Step 4**, else stop.

In our scheme, the activation value of an input pattern at hidden node *m* is computed as the hyperbolic tangent function, it will have a value in the range of [–1, 1]. Steps 1 and 2 of the clustering algorithm find integer representations of all hidden node activation values. A small value for *d* in step 1 indicates that relatively few distinct values for the activation values are sufficient for the network to maintain its accuracy. For example, when *d* = 2, then there could be up to 201 distinct values: −1.00, −0.99, −0.98, …, 0.99, 1.00. For the results reported in this paper, we set the value of *d* = 2.

The array *P* contains the sequence in which the hidden nodes of the network are to be considered. Different ordering sequences usually result in different clusters of activation values. Once a hidden node is selected for clustering, the discretized activation values are sorted in step 5 such that the activation values are in increasing order. The values are clustered based on their distance. We implemented step 6 of the algorithm by first finding a pair of adjacent distinct values with the shortest distance. If these two values can be merged without introducing conflicting data, they will be merged. Otherwise, a pair with the second shortest distance will be considered. This process is repeated until there are no more pairs of values that can be merged. The next hidden node as determined by the array *P* will then be considered.

#### 3.2.4. Rule Extraction (RE) Algorithm

Classification rules are sought in many areas from automatic knowledge acquisition [46] to data mining [47,48] and ANN rule extraction because some of their attractive features. They are explicit, understandable and verifiable by domain experts, and can be modified, extended and passed on as modular knowledge. The proposed rule extraction (RE) algorithm, can be applied to both numeric and discrete data, is composed of three major functions:

- Rule Extraction: This function first initializes the extracted rule list to be empty, and sorts the examples according to example frequency. Then it picks the most frequent occurring example as the base to generate a rule and adds the rule to the list of extracted rules. It then finds all the examples that are covered by the rule and removes them from the example space. It repeats the above process iteratively and continuously adds the extracted rules to the rule list until the examples space becomes empty because all data examples have been covered by the rules extracted and they have all been removed.
- Rule Clustering: Rules are clustered in terms of their class levels. Rules of the same class are clustered together as one group of rules.
- Rule Pruning: Redundant or more specific rules in each cluster are removed. In each of these clusters, more than one rule may cover the same example. For examples, the rule “if (color = green) and (height < 4) then grass” is already contained in a more general rule “if (color = green) then grass”, and thus the rule “if (color = green) and (height < 4) then grass” is redundant. RE eliminates these redundant rules in each cluster to further reduce the size of the best rule list.

A default rule should be chosen to accommodate possible unclassifiable patterns. If rules are clustered, the choice of the default rule is based on clusters of rules. The steps of the rule extraction algorithm are explained as follows:

**Step 1** Extract Rule:
Sort-on-frequency (data-without-duplicates);
*i* = 0;
while (data-without-duplicates is NOT empty){
extract *R_i_* to cover the pattern occurred most frequently;
remove all the patterns covered by *R_i_* ;
*i* = *i*+1;}

The core of this step is a greedy algorithm that finds the shortest rule based on the first order information, which can differentiate the pattern under consideration from the patterns of other classes. It then iteratively extracts shortest rules and remove the patterns covered by each rule until all patterns are covered by the rules.

**Step 2** Cluster Rule:
  Cluster rules according to their class levels. Rules extracted in **Step 1** are grouped in terms of their class levels.
**Step 3** Prune Rule:
  Replace specific rules with more general ones;
  Remove noise rules;
  Eliminate redundant rules;
**Step 4** Check whether all patterns are covered by any rules. If yes then stop, otherwise continue.
**Step 5** Determine a default rule. A default rule is chosen when no rule can be applied to a pattern.

RE exploits the first order information in the data and finds shortest sufficient conditions for a rule of a class that can differentiate it from patterns of other classes. It can extract concise and perfect rules in the sense that the error rate of the rules is not worse than the inconsistency rate found in the original data. The novelty of RE is that the rule extracted by it is order insensitive, *i.e.*, the rules need not be required to fire sequentially.

## 4. Performance Evaluation

This section evaluates the performance of the ESRNN algorithm on a set of well-known benchmark classification problems including diabetes, iris, wine, season, golf playing, and lenses that are widely used in machine learning and data mining research. The datasets representing all the problems were real world data are obtained from [49,50].

### 4.1. Dataset Description

This subsection briefly describes the datasets used in this study. The characteristics of the datasets are summarized in Table 1. The detailed descriptions of the datasets are available in [49,50].

***The diabetes dataset:*** The Pima Indians Diabetes data consists of 768 data pairs with eight attributes normalized between 0 and 1. The eight attributes are number of pregnancies (*A*_1_), plasma glucose concentration (*A*_2_), blood pressure (*A*_3_), triceps skin fold thickness (*A*_4_), Two hour serum insulin (*A*_5_), body mass index (*A*_6_), diabetes pedigree function (*A*_7_), and age (*A*_8_). In this database, 268 instances are positive (output equals 1) and 500 instances are negative (output equals 0).

***The iris dataset:*** This is perhaps the best-known database to be found in the pattern recognition literature. The dataset contains three classes of 50 instances each, where each class refers to a type of Iris plant. Four attributes are used to predict the iris class, *i.e.*, sepal length (*A*_1_), sepal width (*A*_2_), petal length (*A*_3_), and petal width (*A*_4_), all in centimeters. Among the three classes, class 1 is linearly separable from the other two classes, and classes 2 and 3 are not linearly separable from each other. To ease knowledge extraction, we reformulate the data with three outputs, where class 1 is represented by {1, 0, 0}, class 2 by {0, 1, 0}, and class 3 by {0, 0, 1}.

***The wine dataset:*** In a classification context, this is a well-posed problem with “well behaved” class structures. A good dataset for first testing of a new classifier, but not very challenging. These data are the results of a chemical analysis of wines grown in the same region in Italy but derived from three different cultivars. The analysis determined the quantities of 13 constituents found in each of the three types of wines. Number of instances 178, number of attributes 13. All attributes are continuous. This was a three-class problem.

***The season data:*** The season dataset contains discrete data only. There are 11 examples in the dataset, each of which consisted of three-elements. These are weather, tree, and temperature. This was a four-class problem.

***The golf playing data:*** The golf playing dataset contains both numeric and discrete data. There are 14 examples in the dataset, each of which consisted of four-elements. These are outlook, temperature, humidity and wind. This is a two-class problem.

***The lenses data:*** The dataset contains 24 examples and are complete and noise free. The examples highly simplified the problem. The attributes do not fully describe all the factors affecting the decision as to which type, if any, to fit. Number of Instances: 24. Number of Attributes: 4; age, spectacle prescription, astigmatic and tear production rate. All attributes are nominal. This was three-class problem: hard contact lenses, soft contact lenses and not contact lenses.

### 4.2. Experimental Setup

In all experiments, one bias node with a fixed input 1 was used for the hidden and output layers. The learning rate was set between [0.1, 1.0] and the weights were initialized to random values between [–1.0, 1.0]. A hyperbolic tangent function *δ*(*y*) = (*e^y^* −*e*^−^*^y^*)/(*e^y^* +*e*^−^*^y^*) was used as the hidden node activation function and a logistic sigmoid function *σ*(*y*) = 1/ (1 + *e*^−^*^y^*) as the output node activation function.

In this study, all datasets representing the problems were divided into two sets: the training set and the testing set. The numbers of examples in the training set and the testing set was chosen to be the same as those in other works, in order to make the comparison with those works possible. The sizes of the training and testing datasets used in this study are given in Table 2.

### 4.3. Experimental Results

Tables 3–8 show the ANN architectures produced by the ESRNN algorithm and the training epochs over 10 independent runs on a set of benchmark data mining classification problems. The initial architecture has selected before applying the constructive algorithm, which was used to determine the number of nodes in the hidden layer. The intermediate architecture was the outcome of the constructive algorithm, and the final architecture was the outcome of pruning algorithm used in the ESRNN algorithm. It has been seen that ESRNN can automatically determine compact ANN architectures.

**Figure 6.**
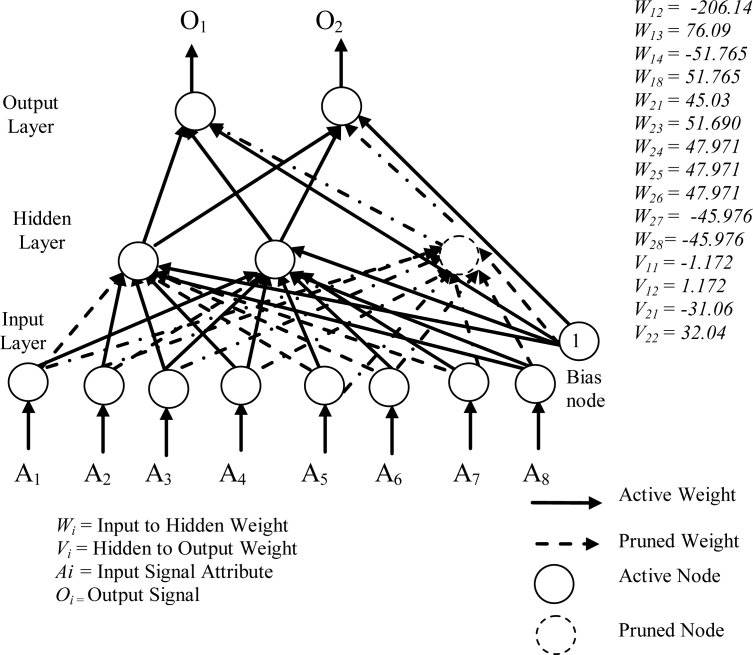
A pruned network for the diabetes data.

Figure 6 shows the smallest of the pruned networks over 10 runs for the diabetes problem. The pruned network was only 2 hidden nodes. No input nodes were pruned by pruning algorithm. One hidden node was pruned, as all the connections to and from this node was pruned. The accuracies on the training data and the testing data have reached 76.30% and 75.52%, respectively. The weight of the connection from the first hidden node to the first output node is −1.172 and to the second output node is 1.172 and the weight of the connection from the second hidden node to the first output node is −31.06 and to the second output node is 32.04. The discrete values found by the heuristic clustering algorithm were −0.968, 0.004 and 0.976.

**Figure 7.**
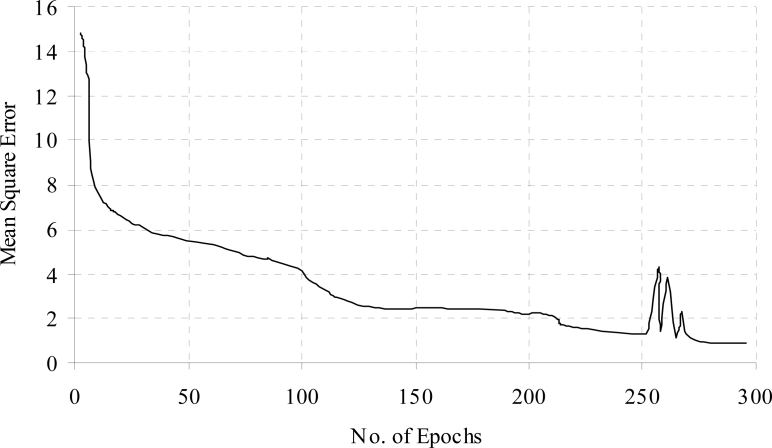
Training time error for the diabetes data.

**Figure 8.**
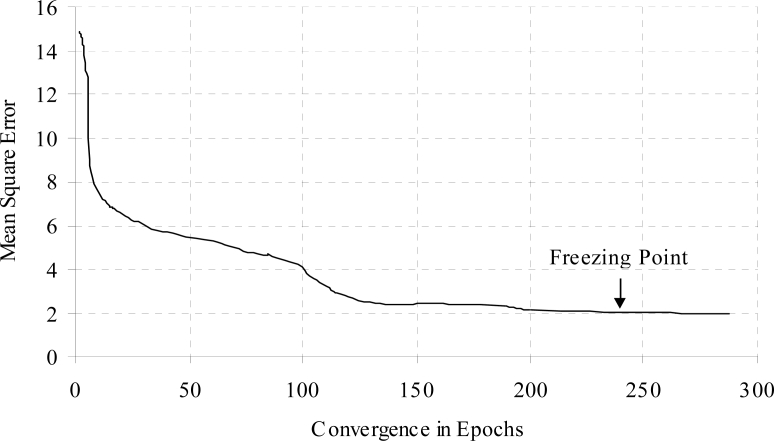
Training time error for the diabetes data with weight freezing.

Figures 7 and 8 show the training time error for diabetes problem. From the Figure 7, it was observed that the training time error decreased and maintained almost constant after some training epochs, it was further decreased when additional hidden nodes were added. The fluctuation was observed due to the connection pruning and finally maintained almost constant value in account of retraining the pruned network.

**Figure 9.**
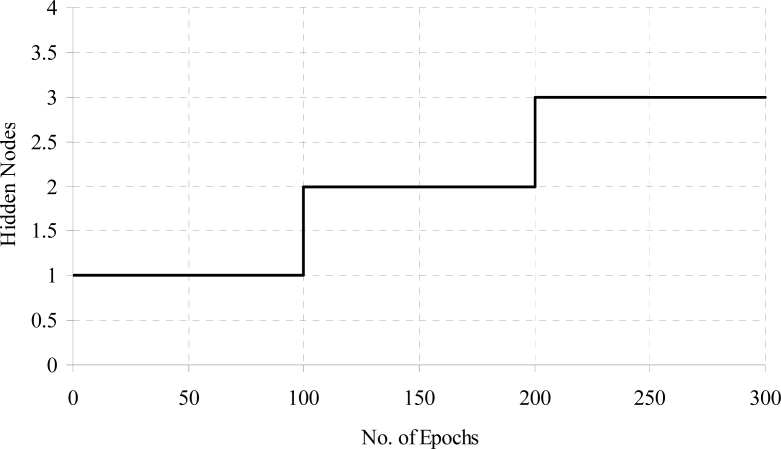
Hidden node addition for the diabetes data.

The training time error for diabetes data with weight freezing is shown in Figure 8. When error is become constant then weight freezing is done. The effects of hidden node addition with increasing the training epochs for diabetes a problem is shown in Figure 9.

### 4.4. Extracted Rules

The number of rules extracted by the ESRNN algorithm and the accuracy of the rules is presented in Table 9, but the visualization of the rules in terms of the original attributes were not discussed. This subsection discusses the rules extracted by ESRNN in terms of the original attributes. The number of conditions per rule and the number of rules extracted have also visualized here.

*The diabetes data*
  Rule 1: If Plasma glucose concentration (*A*_2_) <= 0.64 and Age (*A*_8_) <= 0.69 then tested negative
  Default Rule: tested positive.
*The iris data*
  Rule 1: If Petal-length (*A*_3_) <= 1.9 then iris setosa
  Rule 2: If Petal-length (*A*_3_) <= 4.9 and Petal-width (*A*_4_) <= 1.6 then iris versicolor
  Default Rule: iris virginica.
*The wine data*
  Rule 1: If Input 10 (*A*_10_) <= 3.8 then class 2
  Rule 2: If Input 13 (*A*_13_) >= 845 then class 1
  Default Rule: class 3.
*The season data*
  Rule 1: If Tree (A2) = yellow then autumn
  Rule 2: If Tree (A2) = leafless then autumn
  Rule 3: If Temperature (A3) = low then winter
  Rule 4: If Temperature (A3) = high then summer
  Default Rule: spring.
*The golf playing data*
  Rule 1: If Outlook (A1) = sunny and Humidity >=85 then don’t play
  Rule 2: Outlook (A1) = rainy and Wind= strong then don’t play
  Default Rule: play.
*The lenses data*
  Rule 1: If Tear Production Rate (A4) = reduce then no contact lenses
  Rule 2: If Age (A1) = presbyopic and Spectacle Prescription (A2) = hypermetrope and Astigmatic (A3) = yes then no contact lenses
  Rule 3: If Age (A1) = presbyopic and Spectacle Prescription (A2) = myope and Astigmatic (A3) = no then no contact lenses
  Rule 4: If Age (A1) = pre-presbyopic and Spectacle Prescription (A2) = hypermetrope and Astigmatic (A3) = yes and Tear Production Rate (A4) = normal then no contact lenses
  Rule 5: If Spectacle Prescription (A2) = myope and Astigmatic (A3) = yes and Tear Production Rate (A4) = normal then hard contact lenses
  Rule 6: If Age (A1) = pre-presbyopic and Spectacle Prescription (A2) = myope and Astigmatic (A3) = yes and Tear Production Rate (A4) = normal then hard contact lenses
  Rule 7: If Age (A1) = young and Spectacle Prescription (A2) = myope and Astigmatic (A3) = yes and Tear Production Rate (A4) = normal then hard contact lenses
  Default Rule: soft contact lenses.

Table 9 shows the number of extracted rules and the rules accuracy for a set of benchmark data mining problems. In most of the cases ESRNN produces fewer rules with better accuracy. It was observed that two to three rules were sufficient to solve the problems. The accuracies were 100% for three datasets including season, golf playing, and lenses classification. These datasets have a lower number of examples.

### 4.5. Performance Comparisons

This section compares experimental results of the ESRNN algorithm with the results of other works. The primary aim of this work is not to evaluate ESRNN in order to gain a deeper understanding of rule generation without an exhaustive comparison between ESRNN and all other works. Table 10 compares ESRNN results of the diabetes data with those produced by PMML [29], NN RULES [14], C4.5 [46], NN-C4.5 [51], OC1 [51], and CART [52] algorithms. ESRNN achieved 76.56% accuracy although NN-C4.5 was closest second with 76.4% accuracy. Due to the high noise level, the diabetes problem is one of the most challenging problems in our experiments. ESRNN has outperformed all other algorithms. Table 11 compares ESRNN results of the iris data with those produced by PMML [29], NN RULES [14], DT RULES [14], BIO RE [24], Partial RE [24], and Full RE [24] algorithms. ESRNN achieved 98.67% accuracy although NN RULES was closest second with 97.33% accuracy. Here number of rules extracted by ESRNN and NN RULES are equal. Table 12 shows ESRNN results of the wine data. ESRNN achieved 91.01% accuracy by generating 3 rules. No detailed previous work have found for showing comparison of this dataset.

Table 13 compares the ESRNN results of the season data with those produced by RULES [53] and X2R [25]. All three algorithms achieved 100% accuracy. This is possible because the number of examples is low. ESRNN extracted five rules, whereas RULES extracted seven and X2R six.

Table 14 compares ESRNN results of golf playing data with those produced by RULES [53], RULES-2 [54], and X2R [25]. All four algorithms achieved 100% accuracy because the lower number of examples. Number of extracted rules by ESRNN are three whereas these were eight for RULES and 14 for RULES-2. Finally, Table 15 compares ESRNN results of lenses data with those produced by PRISM [55]. Both algorithms achieved 100% accuracy because the lower number of examples. Number of extracted rules by ESRNN are eight whereas they were nine for PRISM.

## 5. Conclusions

In this paper we have presented a neural network based data mining scheme to mining classification rules from given databases. This work is an attempt to apply the connectionist approach to data mining by extracting symbolic rules similar to that of decision trees. An important feature of the proposed rule extraction algorithm is its recursive nature. A set of experiments was conducted to test the proposed approach using a well defined set of data mining problems. The results indicate that, using the proposed approach, high quality rules can be discovered from the given data sets. The extracted rules are concise, comprehensible, order insensitive, and do not involve any weight values. The accuracy of the rules from the pruned network is as high as the accuracy of the fully connected networks. Experiments showed that this method helped a lot to reduce the number of rules significantly without sacrificing classification accuracy. In almost all cases ESRNN outperformed the others. With the rules extracted by the method introduced here, ANNs should no longer be regarded as black boxes.

## Figures and Tables

**Table 1.** Characteristics of datasets.

**Sl No.**	**Datasets**	**No. of Examples**	**Input Attributes**	**Output Classes**
1	Diabetes	768	8	2
2	Iris	150	4	3
3	Wine	178	13	3
4	Season	11	3	4
5	Golf Playing	14	4	2
6	Lenses	24	4	3

**Table 2.** Sizes of the training and the testing datasets.

**Sl No.**	**Datasets**	**Training Examples**	**Testing Examples**
1	Diabetes	384	384
2	Iris	75	75
3	Wine	89	89
4	Season	6	5
5	Golf Playing	7	7
6	Lenses	12	12

**Table 3.** ANN architectures and the training epochs for the diabetes dataset.

	**Initial Architecture**		**Intermediate Architecture**		**Final Architecture**		**No. of Epochs**
	**No. of Nodes**	**No. of Connections**	**No. of Nodes**	**No. of Connections**	**No. of Nodes**	**No. of Connections**	
Mean	11 (8-1-2)	10	13.1	31	12.1	19.7	306.4
Min	11 (8-1-2)	10	12.3	23	11.9	15	283
Max	11 (8-1-2)	10	13.9	38	13.2	24.4	329

**Table 4.** ANN architectures and the training epochs for the irish dataset.

	**Initial Architecture**		**Intermediate Architecture**		**Final Architecture**		**No. of Epochs**
	**No. of Nodes**	**No. of Connections**	**No. of Nodes**	**No. of Connections**	**No. of Nodes**	**No. of Connections**	
Mean	8 (4-1-3)	7	9	13	9	10.2	198.2
Min	8 (4-1-3)	7	8	8	8	7	185
Max	8 (4-1-3)	7	11	22	10	13.8	220

**Table 5.** ANN architectures and the training epochs for the wine dataset.

	**Initial Architecture**		**Intermediate Architecture**		**Final Architecture**		**No. of Epochs**
	**No. of Nodes**	**No. of Connections**	**No. of Nodes**	**No. of Connections**	**No. of Nodes**	**No. of Connections**	
Mean	17 (13-1-3)	16	18.6	38	17	24.8	215
Min	17 (13-1-3)	16	17.2	18	16	22	198
Max	17 (13-1-3)	16	21	62	21	42	240

**Table 6.** ANN architectures and the training epochs for the season dataset.

	**Initial Architecture**		**Intermediate Architecture**		**Final Architecture**		**No. of Epochs**
	**No. of Nodes**	**No. of Connections**	**No. of Nodes**	**No. of Connections**	**No. of Nodes**	**No. of Connections**	
Mean	8 (3-1-4)	7	8.9	13.1	8.8	11	87
Min	8 (3-1-4)	7	8	7	8	9.1	74
Max	8 (3-1-4)	7	10	14.2	10.5	15	105

**Table 7.** ANN architectures and the training epochs for the golf playing dataset.

	**Initial Architecture**		**Intermediate Architecture**		**Final Architecture**		**No. of Epochs**
	**No. of Nodes**	**No. of Connections**	**No. of Nodes**	**No. of Connections**	**No. of Nodes**	**No. of Connections**	
Mean	7 (4-1-2)	6	8.2	13	7.9	10.5	95.2
Min	7 (4-1-2)	6	7.3	6.1	7.1	6.2	88
Max	7 (4-1-2)	6	9.1	18.2	9.2	13.8	103

**Table 8.** ANN architectures and the training epochs for the lenses dataset.

	**Initial Architecture**		**Intermediate Architecture**		**Final Architecture**		**No. of Epochs**
	**No. of Nodes**	**No. of Connections**	**No. of Nodes**	**No. of Connections**	**No. of Nodes**	**No. of Connections**	
Mean	8 (4-1-3)	7	9.1	13.2	8.7	12	106
Min	8 (4-1-3)	7	8.3	7	8.2	7.8	99
Max	8 (4-1-3)	7	10.4	20.8	11	16	126

**Table 9.** Number of extracted rules and rules accuracies.

**Sl No.**	**Datasets**	**No. of Extracted Rules**	**Accuracy**
1	Diabetes	2	76.56%
2	Iris	3	98.67%
3	Wine	3	91.01%
4	Season	4	100%
5	Golf Playing	3	100%
6	Lenses	8	100%

**Table 10.** Performance comparison of the ESRNN with other algorithms for the diabetes data.

**Dataset**	**Feature**	**ESRNN**	**PMML**	**NN RULES**	**C4.5**	**NN-C4.5**	**OC1**	**CART**
**Diabetes**	**No. of Rules**	2	2	4	–	–	–	–
	**Avg. No. of Conditions**	2	1	3	–	–	–	–
	**Accuracy (%)**	76.56	75	76.32	70.9	76.4	72.4	72.4

**Table 11.** Performance comparison of the ESRNN algorithm with other algorithms for the irish data.

**Dataset**	**Feature**	**ESRNN**	**PMML**	**NN RULES**	**DT RULES**	BIO RE	Partial RE	**Full RE**
**Irish**	**No. of Rules**	3	3	3	4	4	6	3
	**Avg. No. of Conditions**	1	1	1	1	3	3	2
	**Accuracy (%)**	98.67	91.3	97.33	94.67	78.67	78.67	97.33

**Table 12.** Performance of the ESRNN algorithm for the wine data.

**Dataset**	**Feature**	**ESRNN**
**Wine**	**No. of Rules**	3
	**Avg. No. of Conditions**	3
	**Accuracy (%)**	91.01

**Table 13.** Performance comparison of ESRNN with other algorithms for season data.

**Dataset**	**Feature**	**ESRNN**	**RULES**	**X2R**
**Season**	**No. of Rules**	5	7	6
	**Avg. No. of Conditions**	1	2	1
	**Accuracy (%)**	100	100	100

**Table 14.** Performance comparison of ESRNN with other algorithms for golf playing data.

**Dataset**	**Feature**	**ESRNN**	**RULES**	**RULES-2**	**X2R**
**Golf Playing**	**No. of Rules**	3	8	14	3
	**Avg. No. of Conditions**	2	2	2	2
	**Accuracy (%)**	100	100	100	100

**Table 15.** Performance comparison of ESRNN with other algorithm for lenses data.

**Dataset**	**Feature**	**ESRNN**	**PRISM**
**Lenses**	**No. of Rules**	8	9
	**Avg. No. of Conditions**	3	–
	**Accuracy (%)**	100	100
